# Low molecular weight heparin dosing regimens after total joint arthroplasty: a prospective, single-center, randomized, double-blind study

**DOI:** 10.1186/s13018-024-05303-9

**Published:** 2024-11-27

**Authors:** Jingjing Shang, Liangliang Wang, Jinhong Gong, Xinru Liu, Dan Su, Xindie Zhou, Yuji Wang

**Affiliations:** 1 Department of Pharmacy, The Affiliated Changzhou No. 2 People’s Hospital of Nanjing Medical University, Changzhou, China; 2 Department of Orthopedics, The Affiliated Changzhou No. 2 People’s Hospital of Nanjing Medical University, Changzhou, China

**Keywords:** Low molecular weight heparin, Dosing regimens, Total joint arthroplasty, Venous thromboembolism prophylaxis, Ecchymosis, Drainage volume

## Abstract

**Background:**

Low molecular weight heparin (LMWH) has been the standard treatment for preventing venous thromboembolism after total joint arthroplasty. However, the evidence supporting specific LMWH dosing regimens is limited.

**Objectives:**

This study assessed the efficacy and safety of three enoxaparin dosing regimens to prevent venous thromboembolism.

**Methods:**

Participants undergoing hip or knee replacement were randomly assigned to receive 20 mg of enoxaparin 6 h postoperatively (Group A), 40 mg 6 h postoperatively (Group B), or 40 mg 12 h postoperatively (Group C). The primary outcomes included thromboembolic and major bleeding events within 3 months, while the secondary outcomes comprised ecchymosis, wound exudation, drainage volume, allogeneic red blood cell transfusion, and first postoperative day hemoglobin levels.

**Results:**

A total of 536 patients were analyzed. The occurrence of thromboembolic events was comparably low across all groups. Group C exhibited the lowest postoperative ecchymosis rate at 19.3%, significantly less than Group A (32.8%, *p* = 0.004) and Group B (37.7%, *p* < 0.001). Ecchymosis rates were about double in Group A and 1.5 times higher in Group B compared to Group C. Significant differences were also observed in 24-hour and total postoperative drainage volumes, with Group B having higher volumes than the other groups.

**Clinical trial registration:**

This trial was prospectively registered at the China Clinical Trials Registry (registration date: November 14, 2021; registration number: ChiCTR2100053191).

**Conclusion:**

No significant differences in venous thromboembolism rates were seen between the tested enoxaparin dosing regimens after total joint arthroplasty. The 40 mg dose administered 12 h after surgery was associated with reduced postoperative ecchymosis and drainage volumes without an increased thrombosis risk, suggesting it is a safer and more effective option than earlier or lower dosages.

**Graphical Abstract:**

**Figure:**
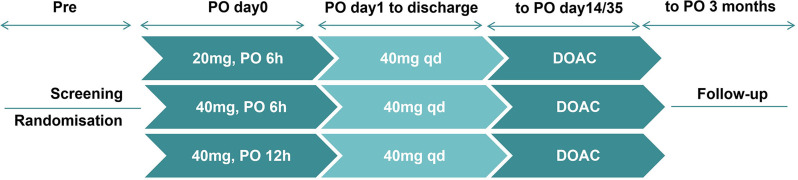

**Supplementary Information:**

The online version contains supplementary material available at 10.1186/s13018-024-05303-9.

## Introduction

Total joint arthroplasty (TJA), including total hip arthroplasty (THA) and total knee arthroplasty (TKA), is widely regarded as an effective treatment for severe joint diseases. Over one million total hip and knee arthroplasties are performed each year in the United States alone [1]. With the aging population and changes in lifestyle, the number of TJAs is increasing annually. While TJA significantly improves the quality of life for patients with joint diseases, it has a notable risk of postoperative complications, particularly venous thromboembolism (VTE), which includes deep vein thrombosis (DVT) and pulmonary embolism (PE). The incidence of VTE following TJA ranges from 1 to 3%, depending on various factors [2].

Preventing VTE following major orthopedic surgery has long been a focus of research and clinical practice [3, 4]. Venous thromboembolism not only causes significant morbidity and mortality but also presents a major financial burden on healthcare systems worldwide. Therefore, effective thromboprophylaxis is essential for mitigating these risks. Low molecular weight heparin (LMWH) is one of the most widely used pharmacological agents for VTE prophylaxis in orthopedic surgery [5]. Multiple clinical guidelines, including those from the American College of Chest Physicians (ACCP) and national guidelines, recommend using LMWH for patients undergoing major orthopedic procedures, such as TJA, to reduce the risk of VTE [6, 7].

Despite the well-documented efficacy of LMWH, the optimal dosing regimen and timing for administration remain contentious. The ACCP recommends LMWH for thromboprophylaxis in patients undergoing major orthopedic surgeries, with at least a 12-hour interval between the administration of LMWH and the procedure [6]. According to the Chinese guidelines for VTE prophylaxis in TJA, LMWH should be administered subcutaneously more than 12 h post-surgery (and 4 h after the removal of the epidural catheter) [7]. However, various studies and clinical practices have reported different approaches. Some experts recommend starting anticoagulation 6–8 h post-surgery or once bleeding has ceased, with a delay of up to 24 h post-surgery for patients at high risk of bleeding [8]. Notably, clinical practices reveal a wide variation in dosing protocols, with many centers using doses ranging from 20 mg to 40 mg. Studies have shown that a moderate dose of LMWH was significantly correlated with reduced thrombosis and all-cause mortality but increased the risk of major bleeding compared to placebo or no treatment [9, 10]. Our preliminary survey on TJA revealed that 21.7% was administered less than 12 h post-surgery, 14.1% was given 12–24 h post-surgery, and the majority of the patients received medication after more than 24 h (64.2%) [11]. In clinical practice, the administration of LMWH lacks a unified standard, highlighting the current lack of consensus in existing protocols.

This study utilized a prospective randomized controlled design to investigate the efficacy of different enoxaparin dosing regimens in preventing VTE in patients undergoing TJA. The three dosing regimens evaluated were 20 mg given at 6 h postoperatively, 40 mg at 6 h postoperatively, and 40 mg at 12 h postoperatively. The primary objective was to examine perioperative LMWH dosing strategies, establish standardized clinical medication protocols, and improve VTE prevention and management in patients undergoing major orthopedic procedures.

## Methods

### Study design and population

This was a prospective, single-center, randomized, double-blind trial registered at the China Clinical Trials Registry (registration date: November 14, 2021; registration number: ChiCTR2100053191). Recruitment for this study began on December 1, 2021, and concluded on July 13, 2023. The patients are randomly assigned if they met the following primary inclusion criteria: age ≥ 18 years; scheduled for primary THA or TKA; bilateral lower extremity venous Doppler ultrasound-negative; no cognitive, linguistic, or intellectual impairments; and they signed informed consent. The principal exclusion criteria were undergoing TJA due to fracture; unable to walk or minimally active; contraindications for anticoagulant or mechanical prophylaxis; preoperative occurrence of DVT or PE; current or long-term use of LMWH, aspirin, or other anticoagulant or antiplatelet medications; and heparin-induced thrombocytopenia. The study was conducted in accordance with the Declaration of Helsinki, the International Conference on Harmonization/Good Clinical Practice Guidelines, and local regulations. Prior to study initiation, the protocol was approved by the Institutional Ethics Committee (approval date: April 19, 2021; approval number: KY026-01), and all patients provided written informed consent before randomization. Additionally, an independent Data and Safety Monitoring Board was established, which reviewed patient safety data every 3 months and reported to the trial steering committee. This committee consisted of independent orthopedic experts, statisticians, pharmacists, and one lay member who oversaw the conduct of the study. This study design adhered to the Consolidated Standards of Reporting Trials (CONSORT) reporting guidelines (see Supplementary Material 1).

### Procedure and randomization

The surgery was performed by two experienced surgeons following discussions in the joint movement group, and all participants received a standard anesthesia protocol formulated by an anesthesiologist. The surgeons’ experience and procedure volume were not significantly different. Both the surgeons and the anesthesiologist were blinded to the randomization. The enrolled patients received either TKA or THA. For TKA, a midline skin incision and standard medial parapatellar approach were used, and for THA, a direct anterior approach was employed. First or second-generation cephalosporins were routinely used preoperatively for prophylaxis and discontinued 24–48 h postoperatively. Vancomycin was used for perioperative prophylaxis in patients allergic to cephalosporins. Consistent with our previous study, patients undergoing THA received a 1 g intravenous infusion of tranexamic acid (TXA) 5–10 min before skin incision, with an additional 1–2 g applied locally during the procedure. For patients undergoing TKA, a tourniquet was used to reduce intraoperative blood loss, with a total duration not exceeding 90 min. The 1 g intravenous infusion of TXA was completed 5–10 min before releasing the tourniquet. No further hemostatic agents were used postoperatively [12]. The wound was closed using a 2 − 0 absorbable suture in layers. The negative pressure drainage tube was routinely left in place postoperatively and removed when drainage was less than 50 mL/day or after 48 h.

**Fig. 1:**
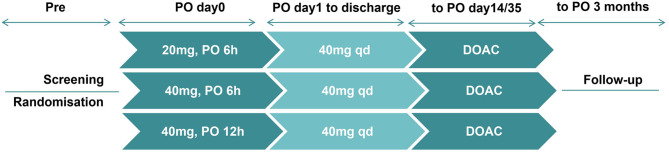
Study Design. Patients were screened according to inclusion criteria and then randomly assigned to receive different anticoagulation regimens, including 20 mg at 6 h post-surgery, 40 mg at 6 h post-surgery, or 40 mg at 12 h post-surgery. From the first day after surgery, 40 mg of enoxaparin was administered once daily until discharge. And the specific administration time was adjusted based on the timing of the first dose. After discharge, prophylaxis with apixaban 2.5 mg twice daily or rivaroxaban 10 mg once daily was used, with the total prophylactic duration extending to 14 days after TKA and 35 days after THA. DOAC, direct oral anticoagulants; PO, postoperative; Pre, preoperative

Researchers used SPSS software to generate a random number table to assign the participants to the treatment groups and retained the assignments in opaque envelopes. The doctors opened these envelopes sequentially as the subjects were enrolled, with each patient assigned a specific random regimen. The prescribing doctor then issued the appropriate medication orders based on the randomization. Neither the patients nor the doctors conducting the postoperative follow-ups were aware of the group assignments. The study design is described in Fig. 1. After initial screening and registration, the eligible patients were randomized in a 1:1:1 ratio to receive different postoperative anticoagulation regimens, including 20 mg at 6 h post-surgery (Group A), 40 mg at 6 h post-surgery (Group B), or 40 mg at 12 h post-surgery (Group C). Enoxaparin was administered by two dedicated nurses who were not involved in the trial and were unaware of the patient outcomes. In the absence of abnormal bleeding, 40 mg of enoxaparin was administered once daily from the first day after surgery until discharge. The specific administration time was adjusted based on the timing of the first dose. After discharge, prevention continued with apixaban 2.5 mg twice daily or rivaroxaban 10 mg once daily. These administrations lasted for 14 days after TKA and for 35 days after THA. During this period, the dosage of anticoagulants was adjusted or discontinued based on the patient’s bleeding status. Patients were clinically monitored twice daily to assess leg swelling, localized tenderness, and leg edema to detect evidence of DVT.

The patients were allowed to stand on the first day after surgery and encouraged to perform full weight-bearing activities using a walker as tolerated. Each patient received immediate postoperative mechanical prophylaxis for 20 min each time, 2 times/day, until discharge. All patients were required to use other medications as prescribed by their doctor throughout the trial. If the timing of medication administration coincided, the patients were instructed to first use the study medication followed by other medications.

### Data acquisition and evaluation

The primary endpoints were thromboembolic and major bleeding events within 3 postoperative months. The predefined secondary endpoints included ecchymosis, wound exudation, wound drainage volume, postoperative transfusion of allogeneic red blood cells, and hemoglobin levels on the first postoperative day. VTE diagnoses were made following the recommendations of the European Society of Cardiology [13]. DVT was diagnosed using compression ultrasonography or venography. Thrombosis in the popliteal vein and/or above was defined as proximal DVT, while thrombosis below the popliteal vein was defined as distal DVT. PE was definitively diagnosed by computed tomography angiography of the pulmonary arteries. Major bleeding and clinically relevant non-major bleeding were defined according to the International Society on Thrombosis and Haemostasis (ISTH) [14]. Anemia was defined by the World Health Organization as a hemoglobin level of less than 12.0 g/dL in women and less than 13.0 g/dL in men [15].

The data collected encompassed demographic variables, such as gender, age, and body mass index, as well as lifestyle factors, including smoking and drinking habits. Additionally, laboratory tests conducted upon admission (including hemoglobin levels, platelet counts, and D-dimer levels), surgical details (including American Society of Anesthesiologists score, type of surgery, duration, intraoperative blood loss, intraoperative transfusion, and TXA usage), postoperative drainage, length of hospital stay, and postoperative hospital stay, were documented. The patients were also assessed for nutritional risk, and the corresponding scores were recorded. Information pertaining to comorbidities linked to heightened risks of thrombotic and bleeding complications, such as diabetes, coronary artery disease, congestive heart failure, peripheral artery disease, and malignancy, was gathered to evaluate the Charlson Comorbidity Index (CCI).

During the trial, healthcare providers meticulously documented the administration of physical prophylaxis, study medication, hemostatic agents, and transfusions, and monitored early and late pain, lower limb swelling, and subcutaneous ecchymosis during the postoperative follow-ups. The researchers reviewed these records during daily visits. Follow-up compression ultrasonography of the bilateral lower extremities was performed on postoperative day 7 and at 1 month and 3 months postoperatively in the outpatient clinic. For participants who did not respond or complete the follow-up assessment, telephone communication was initiated with their designated family members for further evaluation. Patients who declined outpatient follow-up were evaluated remotely using the Wells score. Individuals with a moderate or high probability were recommended to undergo an ultrasound examination at a nearby medical facility.

### Sample size estimation

This randomized controlled trial employed an alpha (α) level of 0.016 to account for multiple comparisons among the three groups. A sample size of 171 per group was determined using Gpower 3.1.9.4, with the alpha set at 0.016, power at 0.9, and the effect size estimated at 0.20 (Cohen’s f) [16]. After accounting for a 10% loss to follow-up and refusal rate, a total of 564 participants (188 per group) were ultimately included in the study.

### Statistical analysis

Statistical analyses were conducted using SPSS 22.0 software. Categorical variables are expressed as counts (percentages) and compared using the χ2 test and Fisher’s exact test. Continuous variables are presented as means (standard deviations) or medians (interquartile ranges) and compared using one-way ANOVA and Kruskal-Wallis H tests. An LSD post hoc test was conducted for individual comparisons between groups. Missing values for variables, such as postoperative D-dimer levels and platelet counts, were estimated using multiple imputations to avoid the statistical test performance reduction and bias caused by the direct exclusion of missing values. Multivariable regression models were employed to adjust for potential confounders such as osteoarthritis and peripheral vascular disease to validate the independent association between enoxaparin dosing regimens and the study endpoints. Regression coefficients (β) or odds ratios (OR) and 95% confidence intervals (CI) were calculated. A p-value of < 0.05 was considered statistically significant.

## Results

**Fig. 2:**
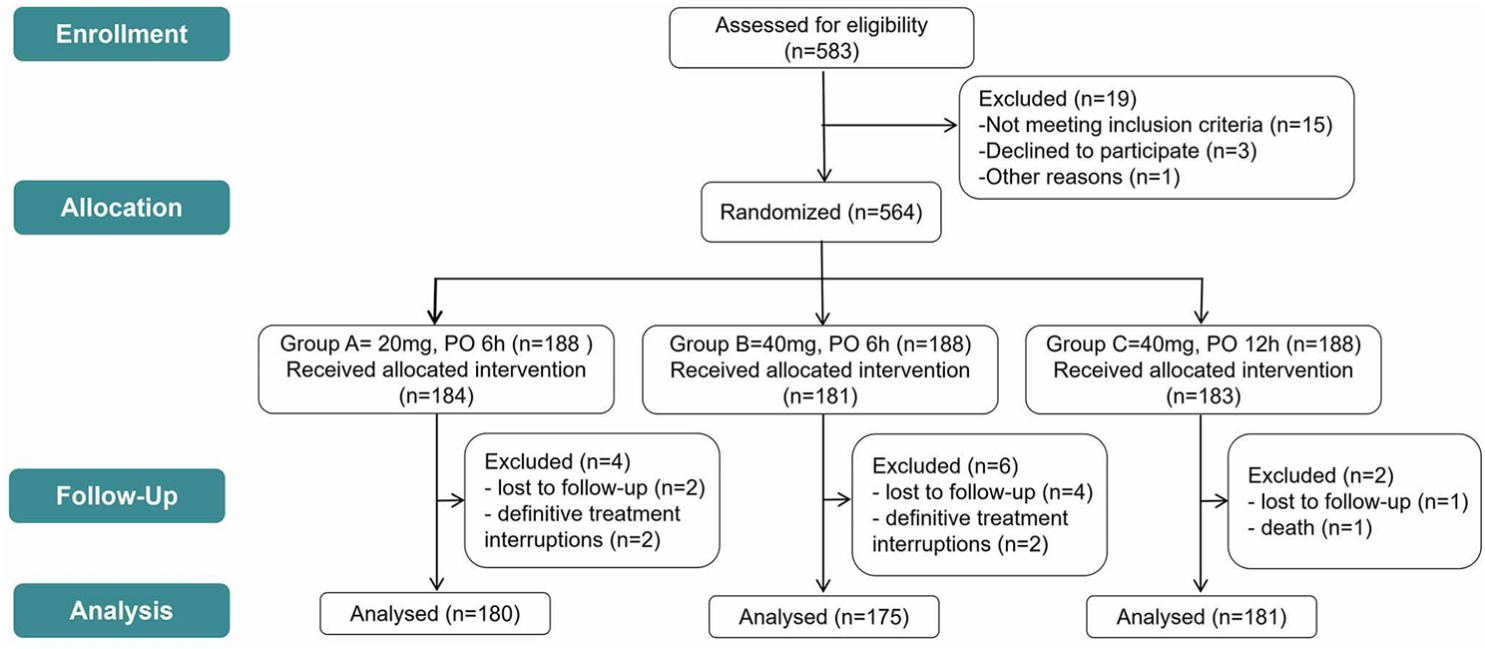
CONSORT diagram of study flow. PO, postoperative.

A total of 564 patients were initially allocated to Groups A, B, and C, with 188 patients in each group. According to the predefined exclusion criteria, 28 patients were excluded from the three groups. Consequently, the final analysis included 536 patients (180 in Group A, 175 in Group B, and 181 in Group C) (Fig. 2). Table 1 presents the baseline characteristics of the enrolled patients. Demographic, clinical, and laboratory data were well-matched across the three groups, except for osteoarthritis and peripheral vascular disease.

**Table 1:** Patient baseline characteristics and demographics

Parameters	Overall	Group A ^a^	Group B ^b^	Group C ^c^	*P* value
	(*n* = 536)	(*n* = 180)	(*n* = 175)	(*n* = 181)	
Demographic data					
Female sex, n(%)	371(69.2)	129(71.7)	117(66.9)	125(69.1)	0.617
Mean age, years, mean(SD)	68.8(9.6)	68.9(9.5)	68.9(9.5)	68.5(9.7)	0.925
Age group, n(%)					0.729
< 70y	272(50.7)	87(48.3)	91(52)	94(51.9)	
≥ 70y	264(49.3)	93(51.7)	84(48)	87(48.1)	
Height, cm , mean(SD)	161.4(7.2)	161.8(7.6)	161.4(7.4)	161.1(6.5)	0.632
Weight, kg , mean(SD)	64.2(10.7)	65.1(10.4)	63.5(11.2)	64.1(10.5)	0.335
Mean BMI, kg/m^2^, mean(SD)	24.6(3.6)	24.9(3.6)	24.4(3.9)	24.6(3.4)	0.408
BMI class, n(%)					
BMI < 18.5	20(3.7)	7(3.9)	9(5.1)	4(2.2)	0.341
BMI ≥ 18.5 and < 25	280(52.2)	89(49.4)	93(53.1)	98(54.1)	0.643
BMI ≥ 25	236(44)	84(46.7)	73(41.7)	79(43.6)	0.638
Smoking, n(%)	92(17.2)	28(15.6)	32(18.3)	32(17.7)	0.773
Drinking, n(%)	91(17)	19(10.6)	34(19.4)	38(21)	0.018
Clinical data					
Type of surgery, n(%)					0.476
Total knee arthroplasty	324(60.4)	111(61.7)	110(62.9)	103(56.9)	
Total hip arthroplasty	212(39.6)	69(38.3)	65(37.1)	78(43.1)	
Mean operative time, min, mean(SD)	106.3(24.5)	104.0(25.4)	105.7(21.4)	109.2(26.2)	0.118
Tranexamic acid, g, median(IQR)	2.1(0.4)	2.1(0.4)	2.1(0.5)	2.1(0.4)	0.737
Intraoperative blood loss, mL, mean(SD)	173.7(129.9)	169.3(115.7)	167.8(119.8)	183.8(150.9)	0.436
Intraoperative transfusion, n(%)	70(13.1)	16(8.9)	25(14.3)	29(16)	0.111
ASA score	2(0)	2(0)	2(0)	2(0)	0.550
Diagnosis, n(%), median(IQR)					
Osteoarthritis	234(43.7)	78(43.3)	101(57.7)	55(30.4)	**<0.001**
Rheumatoid arthritis	29(5.4)	10(5.6)	11(6.3)	8(4.4)	0.735
Osteonecrosis of the femoral head	77(14.4)	26(14.4)	30(17.1)	21(11.6)	0.329
Congenital hip dysplasia	33(6.2)	11(6.1)	15(8.6)	7(3.9)	0.182
Comorbidities, n(%)					
Diabetes mellitus	100(18.7)	30(16.7)	39(22.3)	31(17.1)	0.322
Peripheral vascular disease	50(9.3)	8(4.4)	20(11.4)	22(12.2)	**0.021**
Cardiovascular disease	26(4.9)	0.021(3.3)	9(5.1)	11(6.1)	0.468
Cerebrovascular disease	50(9.3)	14(7.8)	17(9.7)	19(10.5)	0.659
Anemia	24(4.5)	10(5.6)	7(4)	7(3.9)	0.691
Chronic lung disease	48(9)	18(10)	16(9.1)	14(7.7)	0.749
Malignancy	10(1.9)	2(1.1)	2(1.1)	6(3.3)	0.209
Charlson comorbidity index, median(IQR)	0(1)	0(1)	0(1)	0(1)	0.503
NRS 2002 score , median(IQR)	1(1)	1(1)	1(1)	1(1)	0.748
Laboratory data , mean(SD)					
Preoperative hemoglobin, g/L	131.4(16.8)	131.1(16.3)	130.7(16.0)	132.3(18.0)	0.646
Preoperative D-dimer, mg/L	2.1(4.4)	2.1(4.5)	2.2(4.6)	2.0(4.1)	0.907
Preoperative platelet count, *10^9^/L	219.1(68.4)	216.7(67.0)	220.3(62.3)	220.4(75.2)	0.847

^a^ Group A = 20 mg, postoperative 6 h; ^b^ Group B = 40 mg, postoperative 6 h; ^c^ Group C = 40 mg, postoperative 12 h

ASA, American Society of Anesthesiologists; BMI, body mass index; IQR, interquartile range; NRS, nutritional risk screening; SD, standard deviation

**Fig. 3:**
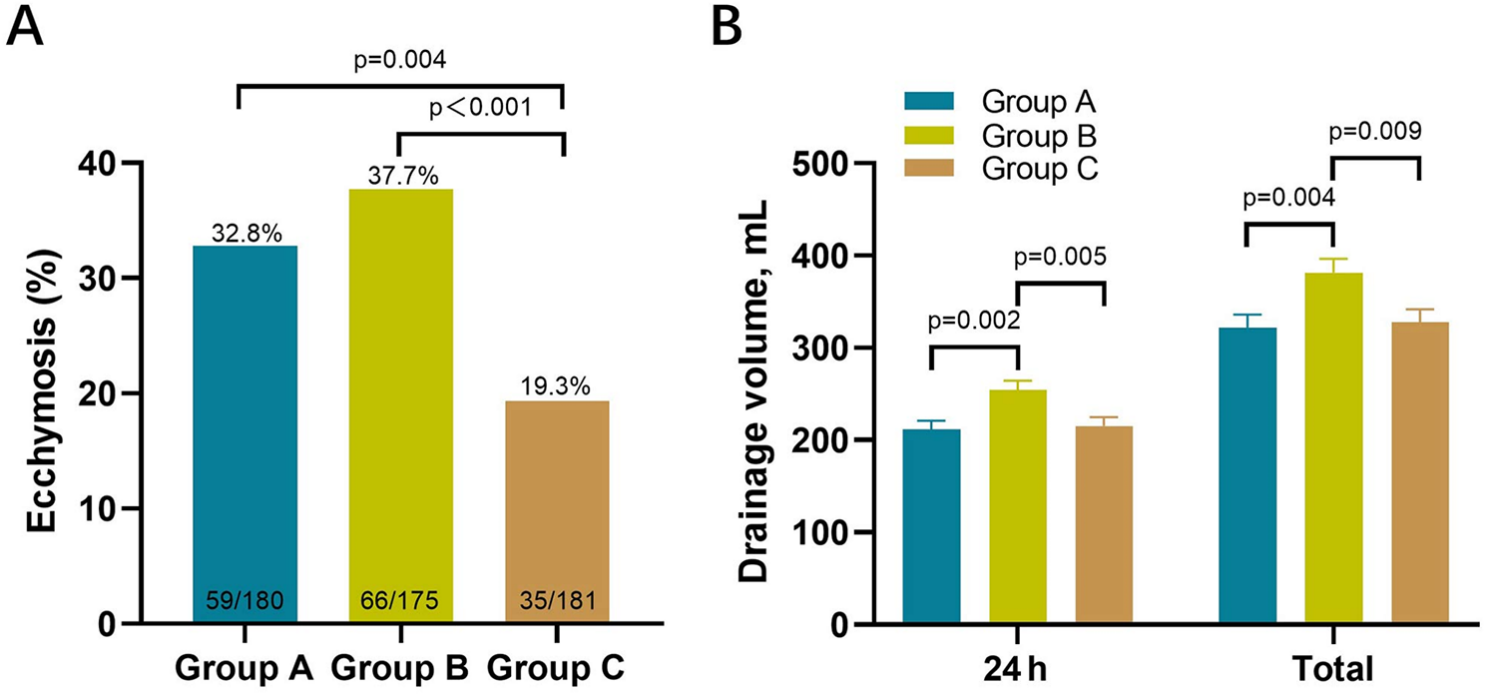
Comparison of ecchymosis and drainage volume (**A**). Ecchymosis (**B**). Drainage volume.Group A = 20 mg, postoperative 6 h; Group B = 40 mg, postoperative 6 h; Group C = 40 mg, postoperative 12 h

As shown in Table 2, during the entire evaluation period, the thrombotic events primarily manifested as distal DVT and muscular vein thrombosis, with all incidences being low and no statistically significant differences between the three groups. No proximal DVT or PE occurred. The bleeding-related events mainly included gastrointestinal bleeding, ecchymosis, and wound exudation. Group C had the lowest incidence of postoperative ecchymosis (19.3%). The post-hoc comparisons revealed that this was significantly lower compared to Group A (32.8%, *p* = 0.004) and Group B (37.7%, *p* < 0.001) (Fig. 3A). Table 3 presents the results of univariate and multivariate logistic regression analyses. The incidence of ecchymosis in Group B was approximately 1.5 times higher compared to Group C (OR: 2.526, 95% CI: 1.564–4.078; *p* < 0.001), and in Group A, it was about one times higher (OR: 2.034, 95% CI: 1.255–3.296; *p* = 0.004). These results remained statistically significant in the multivariate analysis after adjusting for osteoarthritis and peripheral vascular disease (adjusted OR: 1.933–2.290). Similarly, the incidence of gastrointestinal bleeding and wound exudation in Group B showed an increasing trend compared to Groups A and C, but the differences were not statistically significant.

**Table 2:** Primary outcome and secondary outcome

Parameters	Overall	Group A ^a^	Group B ^b^	Group C ^c^	*P* value
	(*n* = 536)	(*n* = 180)	(*n* = 175)	(*n* = 181)	
**Thromboembolic events**, n(%)					
Proximal deep vein thrombosis	0(0)	0(0)	0(0)	0(0)	NA
Distal deep vein thrombosis	14(2.6)	4(2.2)	4(2.3)	6(3.3)	0.766
Intermuscular vein thrombosis	19(3.5)	8(4.4)	5(2.9)	6(3.3)	0.706
**Bleeding events**, n(%)					
Gastrointestinal bleeding	5(0.9)	0(0)	3(1.7)	2(1.1)	0.233
Ecchymosis	160(29.9)	59(32.8)	66(37.7)	35(19.3) ^d^	**<0.001**
Wound exudate	18(3.4)	6(3.3)	9(5.1)	3(1.7)	0.189
Drainage tube removal time, d, median(IQR)	2(0)	2(0)	2(0)	2(0)	0.191
24 h postoperative drainage volume, mL, mean(SD)	226.7(130.7)	211.4(127.5)	254.3(132.0) ^e^	215.2(128.9)	**0.003**
Postoperative total drainage volume, mL, mean(SD)	343.18(196.6)	321.4(197.9)	381.6(198.5) ^f^	327.7(188.9)	**0.006**
Postoperative transfusion, n(%)	43(8.0)	14(7.8)	16(9.1)	13(7.2)	0.784
Overall duration of hospital stay, mean(SD)	10.7(3.4)	10.8(3.7)	10.6(3.0)	10.7(3.5)	0.905
Postoperative duration of hospital stay, mean(SD)	7.3(2.4)	7.5(3.0)	7.3(2.3)	7.1(1.8)	0.448
Hemoglobin on postoperative day 2, g/L, mean(SD)	106.9(15.8)	106.7(15.7)	105.4(14.8)	108.5(16.8)	0.190
D-dimer on postoperative day 2, mg/L, mean(SD)	10.9(8.5)	11.4(9.2)	11.1(8.5)	10.2(7.6)	0.379
D-dimer on postoperative day 5, mg/L, mean(SD)	4.2(2.4)	4.1(2.3)	4.2(2.6)	4.3(2.4)	0.748
Platelet count on postoperative day 2, *10^9^/L, mean(SD)	182.6(58.1)	180.6(54.2)	183.6(54.7)	183.5(64.9)	0.859
Platelet count on postoperative day 5, *10^9^/L, mean(SD)	212.2(66.0)	209.9(63.6)	215.7(63.5)	211.2(70.7)	0.687

^a^ Group A = 20 mg, postoperative 6 h; ^b^ Group B = 40 mg, postoperative 6 h; ^c^ Group C = 40 mg, postoperative 12 h

^d^ Group C vs. Group A, p = **0.004**; Group C vs. Group B, p**<0.001**

^e^ Group B vs. Group A, p = **0.002**; Group B vs. Group C, p = **0.005**

^f^ Group B vs. Group A, p = **0.004**; Group B vs. Group C, p = **0.009**

NA, Not applicable; IQR, interquartile range; SD, standard deviation

**Table 3:** Multivariate regression models for ecchymosis and drainage volume

Variable	Comparison ^a^	Model 1^b^	Model 2 ^c^
Logistics regression			
Ecchymosis	Group C vs. Group A	2.034(1.255–3.296), 0.004	1.933(1.186–3.151), 0.008
	Group C vs. Group B	2.526(1.564–4.078), <0.001	2.290(1.403–3.737),0.001
**Linear regression**			
24 h postoperative drainage volume	Group B vs. Group A	42.953(15.955–69.952),0.002	38.936(11.683–66.188), 0.005
	Group B vs. Group C	39.093(12.132–66.055), 0.005	32.659(5.052–60.267), 0.021
Postoperative total drainage volume	Group B vs. Group A	60.211(19.527-100.895), 0.004	53.560(12.537–94.582), 0.011
	Group B vs. Group C	53.893(13.264–94.522), 0.009	42.927(1.369–84.484), 0.043

^a^ Group A = 20 mg, postoperative 6 h; Group B = 40 mg, postoperative 6 h; Group C = 40 mg, postoperative 12 h

^b^ Model1: unadjusted

^c^ Model2: adjusted for osteoarthritis and peripheral vascular disease

No statistically significant differences were found in the timing of postoperative drainage tube removal among the three groups. However, significant differences were observed in the 24-hour postoperative drainage volume (*p* = 0.003). Group B (254.3 ± 132.0 mL) exhibited significantly higher drainage volume compared to Group A (211.4 ± 127.5 mL, *p* = 0.002) and Group C (215.2 ± 128.9 mL, *p* = 0.005) (Fig. 3B). The volume differences between Groups A and C were not statistically different. Similarly, the total postoperative drainage volume in Group B (381.6 ± 198.5 mL) was significantly higher than that in Group A (321.4 ± 197.9 mL, *p* = 0.004) and Group C (327.7 ± 188.9 mL, *p* = 0.009) (Fig. 3B). Both univariate and multivariate linear regression analyses further substantiated these findings (Table 3). The dosing regimen for Group B caused an increase in 24-hour postoperative drainage of approximately 40 mL and an increase in the total postoperative drainage of nearly 60 mL.

**Fig. 4:**
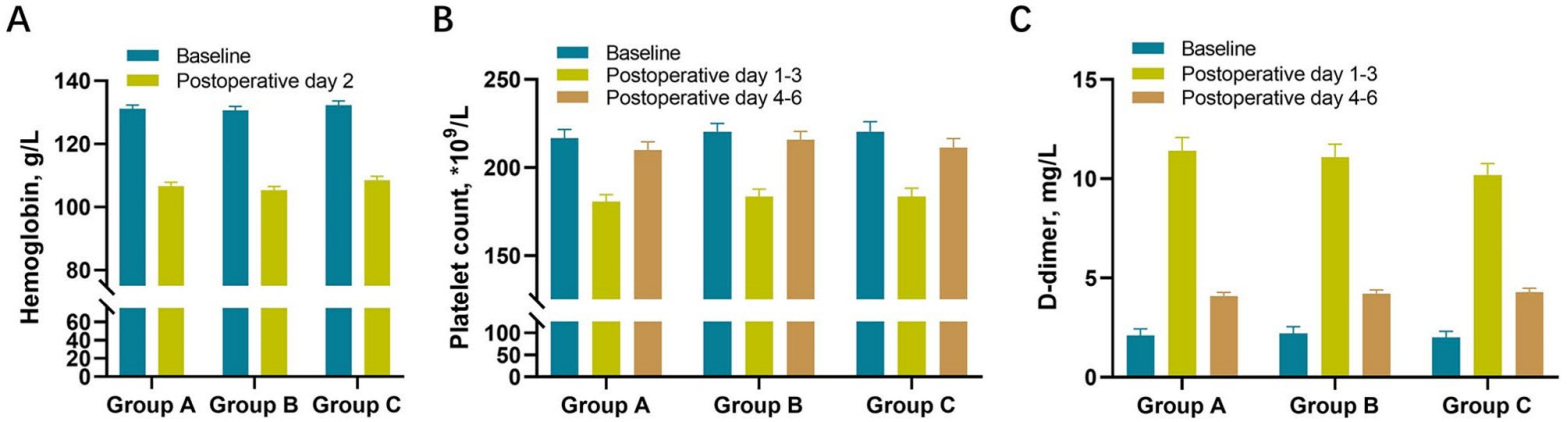
Dynamic changes of laboratory indexes(**A**). Hemoglobin levels (**B**). Platelet counts (**C**). D-dimer levels.Group A = 20 mg, postoperative 6 h; Group B = 40 mg, postoperative 6 h; Group C = 40 mg, postoperative 12 h

Baseline hemoglobin levels, platelet counts, and D-dimer levels were not different among the three groups (Fig. 4A, B, and C). However, compared to baseline values, all three groups showed significant decreases in hemoglobin levels and platelet counts by the second postoperative day and significant increases in D-dimer levels (Fig. 4A, B, and C). By postoperative day 5, the platelet counts had largely returned to preoperative levels, although the D-dimer levels had not yet decreased to the preoperative levels (Fig. 4B and C).

## Discussion

This prospective randomized trial evaluated the impact of different enoxaparin dosing regimens on postoperative thrombotic and bleeding events in patients undergoing TJA. The regimens of 20 mg given 6 h postoperatively, 40 mg given 6 h postoperatively, and 40 mg given 12 h postoperatively were selected following a thorough examination of early data on the use of enoxaparin. Our analysis revealed no significant differences in thromboembolic events among the three cohorts, including proximal DVT, distal DVT, and muscular vein thrombosis. While there were no notable differences in major bleeding events, patients administered a 40 mg dose at 12 h postoperatively exhibited the lowest incidence of ecchymosis compared to the other two groups. In terms of postoperative drainage, the 40 mg dose at 6 h post-surgery resulted in the highest 24-hour and total postoperative drainage volumes. These findings provide evidence for various LMWH dosing strategies.

Several organizations, including the National Institute for Health and Care Excellence (NICE), the ACCP, and the American Academy of Orthopaedic Surgeons (AAOS), recommend medications such as LMWH, rivaroxaban, apixaban, dabigatran, and aspirin for perioperative VTE prevention in TJA [17, 18]. Recent trials and meta-analyses have indicated that aspirin is safe and effective for patients undergoing TJA and may be non-inferior to other major anticoagulants [19, 20]. Migliorini et al. conducted a Bayesian network meta-analysis comparing different anticoagulants used for VTE prevention after THA. The results showed that apixaban demonstrated the best balance between VTE prevention and bleeding control following THA [21]. Longo et al. reported that although selective oral anticoagulants show certain potential in TKA, using these drugs instead of traditional anticoagulants (such as enoxaparin) may carry risks. In particular, the unpredictable off-target effects and the potential for unforeseen side effects could have long-term impacts on patients [22]. Currently, LMWH remains the most commonly used medication for perioperative VTE prevention [4–6]. The action of LMWH involves inhibiting blood coagulation following vascular injury, resulting in an increased incidence of postoperative bleeding. Existing research indicates a significant association between the timing of thromboprophylaxis administration before and after procedures, and the occurrence of bleeding complications in TJA patients [23]. Numerous clinical trials administered LMWH prior to surgery, even 2 h preoperatively. However, the practice of administering LMWH 2 h preoperatively has been linked to a heightened risk of major bleeding [24]. In European clinical settings, LMWH prophylaxis typically commences 12 h prior to elective TJA to maximize efficacy, while in North American settings, initiation generally occurs between 12 and 24 h postoperatively to prioritize safety [25]. The findings of this study suggest that initiating LMWH prophylaxis 12 h postoperatively reduces the occurrence of ecchymosis without elevating the likelihood of postoperative VTE.

Vessel wall injury at the surgical site facilitates the formation of ecchymosis, but the impact of anticoagulants on this process cannot be overlooked. Ecchymosis is a common bleeding complication related to coagulation disorders after TKA, with reported incidence rates as high as 33% [26, 27]. In this study, ecchymosis was defined as subcutaneous hemorrhagic lesions larger than 3 mm, appearing as flat, round, or irregularly shaped blue or purple patches [28]. Postoperative bleeding complications following TJA, including ecchymosis, pose challenges as they may result in more serious complications, such as infections, impaired wound healing, joint dysfunction, and loosening, all of which can impede postoperative recovery and increase postoperative anxiety [29, 30]. In a previously published study, Wang et al. reported an ecchymosis incidence of 27.5% following TKA [31]. The incidence of ecchymosis after TJA in our study was similar to the previously mentioned study, at around 30%. After optimizing the dosing regimen, the overall incidence of ecchymosis was reduced to 19.3%.

Another underexplored issue in major orthopedic surgeries is the optimal dosage of LMWH for preventing VTE. While the package insert recommends adjusting the dosage based on body weight, in clinical practice, a fixed dose is commonly administered for simplicity and ease of use [9]. A meta-analysis by Ruben et al. reported significant differences in the doses of LMWH currently registered and used for thromboprophylaxis. Medium doses of LMWH are significantly associated with a reduction in all-cause mortality, but they also increase the risk of major bleeding compared to placebo or no treatment [10]. The 2012 ACCP guidelines do not provide explicit dosage recommendations, and a review of related literature found that the most commonly administered prophylactic doses are fixed at either 40 mg of enoxaparin once daily or 30 mg twice daily [6]. In our study, administering 40 mg of enoxaparin 12 h postoperatively had the lowest incidence of ecchymosis.

Our study also found that administering 40 mg of enoxaparin 6 h postoperatively was associated with the highest drainage volume at 24 h and total drainage volume. The drainage amount after TJA is the result of multiple factors, including the type of surgery, hemostatic measures, the use of anticoagulants and hemostatic agents, patient age, and underlying conditions [32–34]. In our baseline data, no statistically significant differences were found among the three groups regarding surgery type, the use of TXA, age, and other factors. Previous studies have indicated that different drainage methods are key factors influencing postoperative bleeding in TJA. Jeon et al. explored the optimal timing for releasing temporary drain clamping after TKA [35]. Their results showed that a 3-hour temporary clamp duration effectively reduced bleeding and transfusion requirements while also alleviating acute pain and promoting recovery of joint mobility. Recently, Sa-ngasoongsong et al. demonstrated that the use of low-dose intra-articular TXA combined with prolonged drain clamping (12 h) is a safe and effective blood conservation technique that does not increase systemic absorption [36]. Additionally, there is ongoing debate regarding the routine use of a drainage tube postoperatively [37]. The 2024 World Expert Meeting on Arthroplasty clearly stated that the routine use of surgical drains after primary total knee and hip arthroplasty is not recommended. Based on high-level evidence, 83.52% of experts support this conclusion [38]. Additionally, the role of the tourniquet in knee arthroplasty remains controversial. Migliorini et al. conducted a Bayesian network meta-analysis on the effect of tourniquet use in TKA. The results showed that the group without a tourniquet had the lowest incidence of DVT and demonstrated the best outcomes in pain control, range of motion, and knee function scores compared to the group in which a tourniquet was used [39]. In our study, a tourniquet was used during TKA to reduce perioperative bleeding and shorten the surgical time, and a negative pressure drain was routinely placed postoperatively for all patients to minimize intergroup differences. After adjusting for admission diagnoses of osteoarthritis and comorbid peripheral vascular disease, the administration of 40 mg at 6 h postoperatively resulted in an increase in drainage volume of approximately 35 mL at 24 h and nearly 50 mL in total drainage volume.

Our study had several limitations. First, as it was a single-center study, the generalizability of our findings may be limited, and further regional studies are needed to support our conclusions. Second, we did not differentiate the severity of ecchymosis, which might have revealed different levels of severity. Third, the study was affected by the COVID-19 pandemic, leading to some patients dropping out of the follow-up because they preferred not to visit the hospital for ultrasound examinations to minimize their risk of infection. Fourth, the use of a drainage tube may result in increased postoperative ecchymosis and blood loss. Despite these limitations, our study holds significant clinical relevance. This trial was prospectively designed, randomized, double-blinded, and monitored by an independent Data and Safety Monitoring Board, providing a strong indication that the enoxaparin regimen of 40 mg administered 12 h postoperatively is both effective and safe. Additionally, we conducted a 90-day follow-up for all patients to ensure that no clinically significant events were missed. Although this study focused on patients undergoing TJA, patients with femoral fractures were excluded as the clinical needs and management strategies for this subgroup of patients differ significantly. Patients with femoral fractures should be managed through a multidisciplinary approach, including early surgery and appropriate thromboprophylaxis, to reduce the incidence of VTE and improve postoperative care [40].

### Conclusion

In summary, no statistically significant difference in the efficacy of VTE prevention between LMWH dosing regimens of 20 mg or 40 mg 6 h postoperatively and 40 mg 12 h postoperatively in patients undergoing total hip or knee arthroplasty. However, 40 mg administered 12 h postoperatively was associated with a decrease in postoperative ecchymosis and drainage volume.

## Electronic supplementary material

Below is the link to the electronic supplementary material.

Supplementary Material 1

## Data Availability

As data contain personal health information of individuals, and data were strictly used under license for the current study. The datasets used and/or analysed during the current study are available from the corresponding author on reasonable request.

## Abbreviations

ACCP: American College of Chest Physicians
ASA: American Society of Anesthesiologists
CCI: Charlson Comorbidity Index
CONSORT: Consolidated Standards of Reporting Trials
DVT: Deep vein thrombosis
ISTH: International Society on Thrombosis and Haemostasis
LMWH: Low molecular weight heparin
PE: Pulmonary embolism
THA: Total hip arthroplasty
TJA: Total joint arthroplasty
TKA: Total knee arthroplasty
TXA: Tranexamic acid
VTE: Venous thromboembolism
